# Multi-omics integration and immune profiling identify possible causal networks leading to uterine microbiome dysbiosis in dairy cows that develop metritis

**DOI:** 10.1186/s42523-024-00366-9

**Published:** 2025-01-09

**Authors:** S. Casaro, J. G. Prim, T. D. Gonzalez, F. Cunha, A. C. M. Silva, H. Yu, R. S. Bisinotto, R. C. Chebel, J. E. P. Santos, C. D. Nelson, S. J. Jeon, R. C. Bicalho, J. P. Driver, Klibs N. Galvão

**Affiliations:** 1 Department of Large Animal Clinical Sciences, University of Florida, Gainesville, FL 32610 USA; 2 Department of Clinical Sciences, Auburn University, Auburn, AL 36849 USA; 3 Department of Animal Sciences, University of Florida, Gainesville, FL 32610 USA; 4 D. H. Barron Reproductive and Perinatal Biology Research Program, University of Florida, Gainesville, FL 32610 USA; 5 Department of Veterinary Biomedical Sciences, Long Island University, Brookville, NY 11548 USA; 6 FERA Diagnostics and Biologicals, College Station, TX 77845 USA; 7 Division of Animals Sciences, University of Missouri, Columbia, MO 65211 USA

**Keywords:** Microbiome, Metabolome, Immune dysregulation, Multi-omics, Causal networks

## Abstract

**Background:**

Cows that develop metritis experience dysbiosis of their uterine microbiome, where opportunistic pathogens overtake uterine commensals. An effective immune response is critical for maintaining uterine health. Nonetheless, periparturient cows experience immune dysregulation, which seems to be intensified by prepartum over-condition. Herein, Bayesian networks were applied to investigate the directional correlations between prepartum body weight (BW), BW loss, pre- and postpartum systemic immune profiling and plasma metabolome, and postpartum uterine metabolome and microbiome.

**Results:**

The Bayesian network analysis showed a positive directional correlation between prepartum BW, prepartum BW loss, and plasma fatty acids at parturition, suggesting that heavier cows were in lower energy balance than lighter cows. There was a positive directional correlation between prepartum BW, prepartum systemic leukocyte death, immune activation, systemic inflammation, and metabolomic changes associated with oxidative stress prepartum and at parturition. Immune activation and systemic inflammation were characterized by increased proportion of circulating polymorphonuclear cells (PMN) prepartum, B-cell activation at parturition, interleukin-8 prepartum and at parturition, and interleukin-1β at parturition. These immune changes together with plasma fatty acids at parturition had a positive directional correlation with PMN extravasation postpartum, which had a positive directional correlation with uterine metabolites associated with tissue damage. These results suggest that excessive PMN migration to the uterus leads to excessive endometrial damage. The aforementioned changes had a positive directional correlation with *Fusobacterium*, *Porphyromonas*, and *Bacteroides* in cows that developed metritis, suggesting that excessive tissue damage may disrupt physical barriers or increase substrate availability for bacterial growth.

**Conclusions:**

This work provides robust mechanistic hypotheses for how prepartum BW may impact peripartum immune and metabolic profiles, which may lead to uterine opportunistic pathogens overgrowth and metritis development.

**Supplementary Information:**

The online version contains supplementary material available at 10.1186/s42523-024-00366-9.

## Background

Metritis affects 25% of dairy cows [1], affecting animal wellbeing [2] and performance, which leads to reduced dairy farm profitability [2–4]. The uterine microbiome from cows that develop metritis and those that remain healthy do not differ until 2 days postpartum [5, 6], after which opportunistic pathogens, such as *Fusobacterium*, *Porphyromonas*, and *Bacteroides* overtake the uterine commensals [5–8]. It is not clear why these opportunistic pathogens thrive and cause metritis; however, the metabolic and immune changes associated with parturition and the onset of lactation seem to play an important role in metritis development [9, 10]. Around parturition, most dairy cows experience a period of negative nutrient balance, which is more pronounced in overconditioned cows [11] and in cows that develop metritis [4]. This period of pronounced negative nutrient balance is associated with lesser blood calcium [12], lesser amino acids [13], and greater circulating fatty acids concentration [10] in cows that develop metritis when compared with cows that do not develop metritis. These metabolic changes have been associated with persistent systemic inflammation, oxidative stress, cellular damage, and immune dysregulation [9, 10]. Studies to date have only evaluated how a limited number of metabolites affect the systemic immune response in cows that develop metritis, which does not fully represent the wide variety of metabolic pathways involved in immune processes [14]. Furthermore, vascular degeneration that occurs shortly after parturition in dairy cows allows for the exchange of metabolites between blood and uterus [15, 16], thereby raising the potential for blood metabolites to influence the uterine microbiome, or microbial-derived uterine metabolites to influence the systemic immune response. Either of these mechanisms could affect bacterial proliferation in the uterus. We have previously demonstrated that the peripartum plasma [13] and uterine [6] metabolome and immune response [10] differ between cows that develop metritis and those that do not; however, the interplay between plasma and uterine metabolome, systemic immune response, and the uterine microbiome has not been investigated. Therefore, the hypothesis of the current study was that plasma and/or uterine metabolites promote opportunistic pathogenic bacterial overgrowth in cows that develop metritis either directly or indirectly by regulating the systemic immune response. Thus, the objective of the current study was to apply directionality networks to infer possible causal relationships between the uterine and plasma metabolome, systemic immune response, and the uterine microbiome to advance the understanding of metritis development.

## Methods

**Fig. 1:**
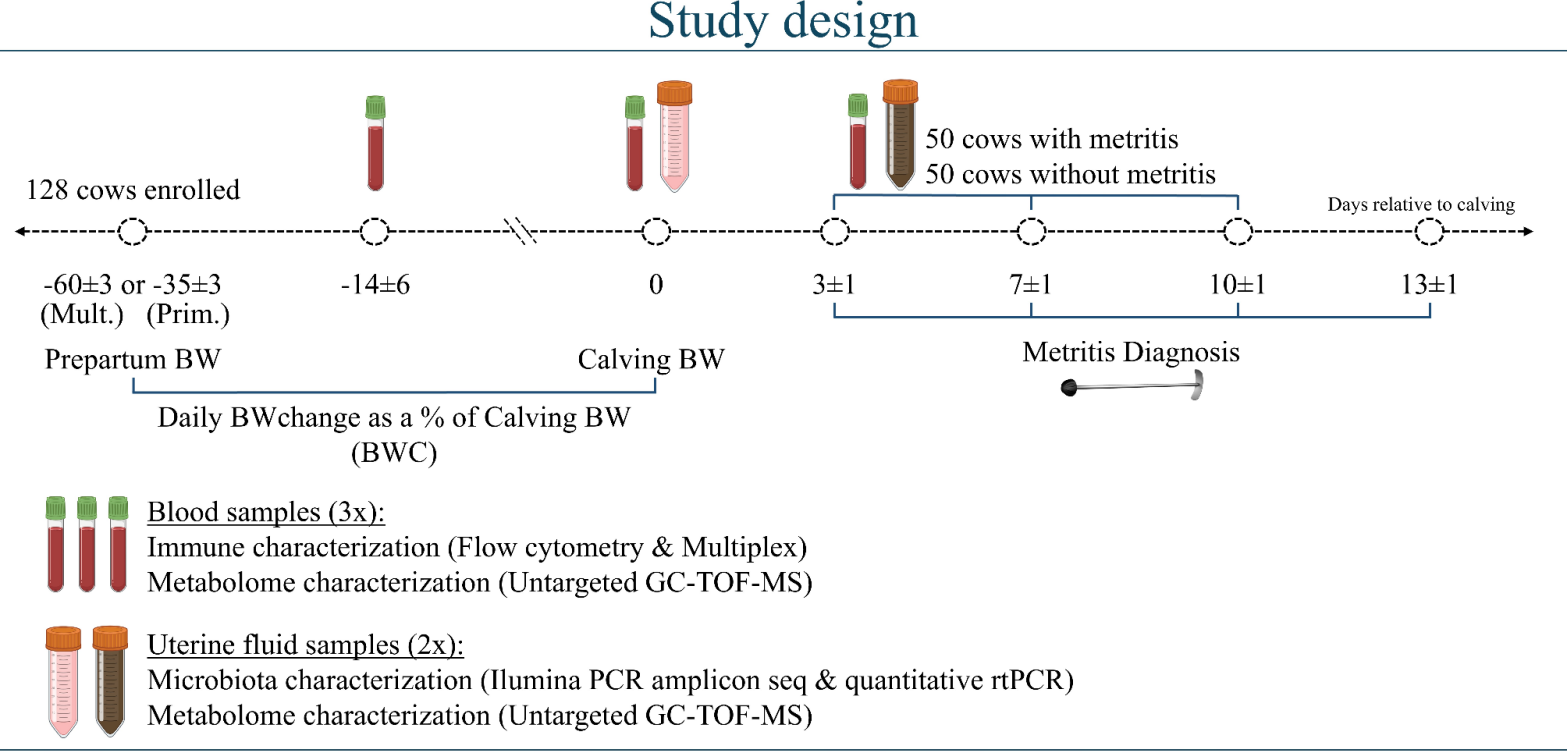
Study design. Primigravid (Prim.) cows were weighed twice at 240 d of gestation and the mean value was used as their prepartum body weight (ppBW). Multiparous (Mult.) cows were weighted at -2, -1, and 0 days relative to dry-off from the lactation preceding enrolment. The 3 measurements were used to generate a mean ppBW. On 0, 1, and 2 days relative to calving primiparous and multiparous cows were weighted. The 3 measurements were used to generate a mean calving BW (cBW). To only account for the body weight change (BWC) related to tissue accretion or mobilization, the weight of the gravid uterus prepartum and the weight of the empty uterus right after parturition were subtracted from the ppBW and cBW, respectively. The weight of the gravid uterus and empty uterus were calculated using NASEM (2021) equations. After excluding the weight of the gravid uterus from the ppBW and the weight of the empty uterus from the cBW, the BWC prepartum was calculated as the difference between cBW and ppBW divided by the number of days between measurements. To account for any differences in BWC associated with frame, BWC then was calculated as a percentage of cBW. Uterine discharge was evaluated using a Metricheck device (Metricheck, Simcro, New Zealand) at 3 ± 1, 7 ± 1, 10 ± 1 and 13 ± 1 days after calving. Cows diagnosed with metritis up to day 10 were considered cases for this study. There were 52 metritis cases, and 10, 23, and 19 cows were diagnosed with metritis on day 3, 7, and 10, respectively. Two cows were excluded because of several missing values for Bayesian networks on the day of metritis diagnosis. Control cows (*n* = 50) that did not develop metritis were matched with 50 cows that developed metritis, hence, 100 cows were used for bioinformatic and statistical analyses. All cows had blood collected prepartum (14 ± 6 d before calving), at calving (first 24 h after calving), and at the day of metritis diagnosis (7 ± 2 d after calving). All cows had uterine fluid collected at calving and at diagnosis of metritis. Blood samples were used for flow cytometry, multiplex, and untargeted gas chromatography mass spectrometry (GC-TOF-MS). Flow cytometry and multiplex were performed to assess the systemic immune profile and activation status. Gas chromatography mass spectrometry was performed to assess the systemic metabolomic profile. Uterine fluid samples were used for untargeted GC-TOF-MS and 16 S rRNA gene amplification by PCR. Gas chromatography mass spectrometry was performed to assess the uterine metabolomic profile and the 16 S rRNA gene was amplified by PCR to assess the uterine microbiome

This case-control observational study was conducted at the University of Florida Dairy Unit from September 2019 to March 2020. The cows used for the current study were a subset of cows used in our previous studies [6, 9, 10]. All procedures involving cows were approved by the Institutional Animal Care and Use Committee of the University of Florida; protocol number 201,910,623. The study design can be found in Fig. 1.

Briefly, cows were enrolled at 240 d of gestation. Primigravid cows were weighed twice at 240 d of gestation and the mean value was used as their prepartum body weight (ppBW). Multiparous cows’ BW were collected from the on-farm computer software (Afifarm management program, Afimilk Ltd, Kibbutz Afikim, Israel). Three consecutive weights were collected at -2, -1, and 0 days relative to dry-off from the lactation preceding enrolment. The 3 measurements were used to generate a mean ppBW. On 0, 1, and 2 days relative to parturition weights were collected from the on-farm computer software from primiparous and multiparous cows. The 3 measurements were used to generate a mean calving BW (cBW). To only account for the body weight change (BWC) related to tissue accretion or mobilization, the weight of the gravid uterus prepartum and the weight of the empty uterus right after parturition were subtracted from the ppBW and cBW, respectively. The weight of the gravid uterus and empty uterus were calculated using NASEM (2021) [18] equations as follows:

$$\:Gravid\:Uterine\:Weight=\left[\left(Calf\:BW\:\times\:1.825\right)\times\:\:{e}^{-\left[0.0243-\left(0.0000245\:\times\:day\:of\:gestation\right)\right]\:\times\:\:\left(280-day\:of\:gestation\right)}\right]\:\:\:\:\:\:$$

$$\:Empty\:Postpartum\:Uterine\:Weight=\left(Calf\:BW\:\times\:0.2288\right)$$

After excluding the weight of the gravid uterus from the ppBW and the weight of the empty uterus from the cBW, the BWC prepartum was calculated as the difference between cBW and ppBW divided by the number of days between measurements. To account for any differences in BWC associated with frame, BWC then was calculated as a percentage of cBW. Uterine discharge was evaluated using a Metricheck device (Metricheck, Simcro, New Zealand) at 3 ± 1, 7 ± 1, 10 ± 1 and 13 ± 1 days after parturition using a 5-point scale as previously described [10]: 1 = not fetid normal lochia, viscous, clear, red, or brown; 2 = cloudy, pink, red, or brown mucoid discharge with flecks of pus; 3 = not fetid, pink red or brown mucopurulent discharge with < 50% pus; 4 = not fetid, pink, red or brown purulent discharge with ≥ 50% pus; 5 = fetid red-brownish, watery discharge. Cows with a uterine discharge score of 5 in at least one examination were classified as having metritis and cows with a discharge score ≤ 4 were classified as not having metritis. Cows diagnosed with metritis up to day 10 were considered cases for this study. There were 52 metritis cases, and 10, 23, and 19 cows were diagnosed with metritis on day 3, 7, and 10, respectively. Two cows were excluded because of several missing values for Bayesian networks on the day of metritis diagnosis. Control cows (*n* = 50) that did not develop metritis were matched with 50 cows that developed metritis, hence, 100 cows were used for bioinformatic and statistical analyses. An attempt was made to pair cows that did and did not develop metritis according to the day of metritis diagnosis, although it was not always possible. Of the control cows, 10, 30, and 10 cows were sampled on day 3, 7, and 10, respectively. Of these 50 cows, 14, 16, 13, and 7 cows had a uterine discharge score of 1, 2, 3, and 4, respectively. All cows had blood collected prepartum (14 ± 6 d before parturition), at parturition (first 24 h after parturition), and at the day of metritis diagnosis (7 ± 2 d after parturition). All cows had uterine fluid collected at parturition and at diagnosis of metritis. Blood samples were used for flow cytometry, multiplex, and untargeted gas chromatography mass spectrometry. Flow cytometry and multiplex were performed to assess the systemic immune profile and activation status. Gas chromatography mass spectrometry was performed to assess the systemic metabolomic profile. Uterine fluid samples were used for untargeted gas chromatography mass spectrometry and 16 S rRNA gene amplification by PCR. Gas chromatography mass spectrometry was performed to assess the uterine metabolomic profile and the 16 S rRNA gene was amplified by PCR to assess the uterine microbiome. Full details on animal data, immune profiling, metabolome and microbiome data can be found in Supplemental Materials and Methods.

### Data pre-processing

Data pre-processing was performed using R [17]. Data included in the study was published elsewhere [6, 9, 10]. Systemic immune data [10] and plasma metabolome [9] consisted of three timepoints: prepartum, parturition, and the day of metritis diagnosis. Uterine microbial estimated counts and uterine metabolome [6] consisted of two timepoints: parturition and the day of metritis diagnosis. Data on ppBW and BWC was previously published together with immune data [10]. Variables with missing values were excluded from further analysis. For systemic immune data, only variables with a *P* ≤ 0.10 to the effect of metritis in our previous study [10] and without missing values were selected for further analysis. The variables used herein for prepartum were Live %, Monocytes %, polymorphonuclear cells (PMN) %, B-cell activation (CD62L negative), interleukin (IL)-8, and interferon (IFN)-y. For parturition the variables were Live %, B-cell activation, Monocyte activation (increase in CD62L median fluorescence intensity [MFI]), IL-1β, IL-8, and IFN-γ. For the day of diagnosis, the variables were Live %, Singlets %, Monocytes %, PMN %, Monocyte activation, Monocyte Major Histocompatibility Complex Class II (MHCII) %, PMN activation (increase in CD62L MFI), B-cell activation, and T helper activation (reduction in CD62L MFI).

To eliminate any possible effect of parity from the analyses, all variables from the datasets were divided by parity group (primiparous and multiparous), log transformed and auto scaled within parity group, and then merged back together.

### Latent variables identification and significance

Latent variables identification and significance were performed using the mixOmics R package [18]. Because microbiome and metabolome data are composed of highly correlated variables, principal component analysis (PCA) was applied to microbiome and metabolome data for orthogonal latent variables identification.

For the five metabolomics datasets, sparse PCA (sPCA) via regularized single value decomposition [19] was applied using the ‘spca’ function of the mixOmics package. Briefly, the sPCA from MixOmics is based on singular value decomposition, which is appropriate when dealing with large datasets where not all variables are likely to be equally important. The sparsity is achieved via LASSO penalization, such that latent variables are no longer a linear combination of all original variables but are a linear combination of a subset of variables that maximize the captured variance. Five latent variables were created for each dataset. For each latent variable, optimal number of variables were selected using 3-fold cross-validation with 10 repetitions. This approach was taken to prevent overfitting by dividing the data into three subsets (folds), training the model on two folds while validating it on the third, and repeating the process 10 times for robustness. This ensures that the number of selected variables is optimal and generalizable, providing a more reliable model that performs well across different portions of the data.

For the uterine microbiome at parturition and at the day of diagnosis a PCA was applied using the ‘pca’ function of the mixOmics package selecting the opportunistic pathogenic bacteria with the greatest relative abundance [6, 8] and estimated counts [6] in cows with metritis compared with cows without metritis. These were *Fusobacterium*, *Porphyromonas*, and *Bacteroides*. One latent variable was created for parturition and one for the day of metritis diagnosis. The rationale behind this decision was to observe how different immune and metabolic variables affected the growth of these bacteria since parturition.

Latent variables significance between cows that developed metritis and cows that did not develop metritis were assessed by *t*-tests using the t.test function of the stats R package [17]. Statistical significance was considered if *P* ≤ 0.10.

### Bayesian networks

Bayesian network analysis allows for both directionality and probabilistic inference, enabling us to model the conditional dependencies between variables in a clear and interpretable manner. The conditional dependencies can be interpreted as potential causal relationships among variables. Nonetheless, care must be taken in interpreting these directional relationships as causal because not all the assumptions for causality can be met in an observational study such as that there are no unobserved external variables affecting the variables in the network [20, 21]. Bayesian network analysis was performed using the bnlearn R package [20]. Highly correlated variables (> 0.80 or < -0.80) were excluded to avoid multicollinearity. Prepartum B-cell activation was excluded because they were highly correlated with parturition (Pearson correlation coefficient *r* = 0.83) and diagnosis (*r* = 0.82) B-cell activation. A total of 20 immune variables, 14 metabolomic latent variables, 2 microbiome latent variables, and 2 metadata variables were used to study their probabilistic relationships. Structure learning was performed using hill climbing, tabu, and max–min hill climbing algorithms. A blacklist based on prior biological knowledge was added to the structure learning algorithms to establish a temporal logic model such that nodes from the day of diagnosis were not allowed to predict nodes at parturition or prepartum, nodes at parturition were not allowed to predict nodes prepartum, and prepartum nodes were not allowed to predict nodes at the day of diagnosis. Each algorithm was bootstrapped 1,000 times and mean and median Bayesian information criterion (BIC) scores were extracted. The algorithm with the mean and median larger BIC scores was chosen since bnlearn re-scales the BIC scores by − 2, meaning that the larger the BIC scores, the better the Bayesian network explains the data.

The best algorithm was then bootstrapped 1,000 times to estimate the uncertainty of the edge’s strength and the direction of the network as previously described [21, 22]. Edges showing presence in at least 60% (strength) among all the 1,000 models were kept in the Bayesian network through model averaging. Model estimates were extracted for each permutation to calculate a mean estimate with a 95% confidence interval for each edge to understand if the parent node was positively or negatively predicting the child node.

Once the network was created, it was exported and edited using Metscape 2 [23] within the CytoScape 3.8 platform. Only edges and nodes that were part of the network connected to the latent variable composed of opportunistic pathogenic bacteria at the day of diagnosis were kept in the final network for ease of interpretation.

## Results

### Latent variables identification

Within each metabolomic dataset, five principal components (PC) were generated as latent variables. These components were sequentially labeled from PC1 to PC5, representing distinct dimensions of variation captured from each dataset. For the microbiome datasets, one PC at parturition and one PC at metritis diagnosis were generated. Only PCs that remained in the Bayesian network will be described. Full details about each PC can be found in SI Results. Each dataset explained variation by each PC can be found in Supplemental Table S1. Loadings for each plasma and uterine PCs can be found in Supplemental Table S2 and Supplemental Table S3, respectively. Association between each PC and metritis can be found in Supplemental Table S4.

**Fig. 2:**
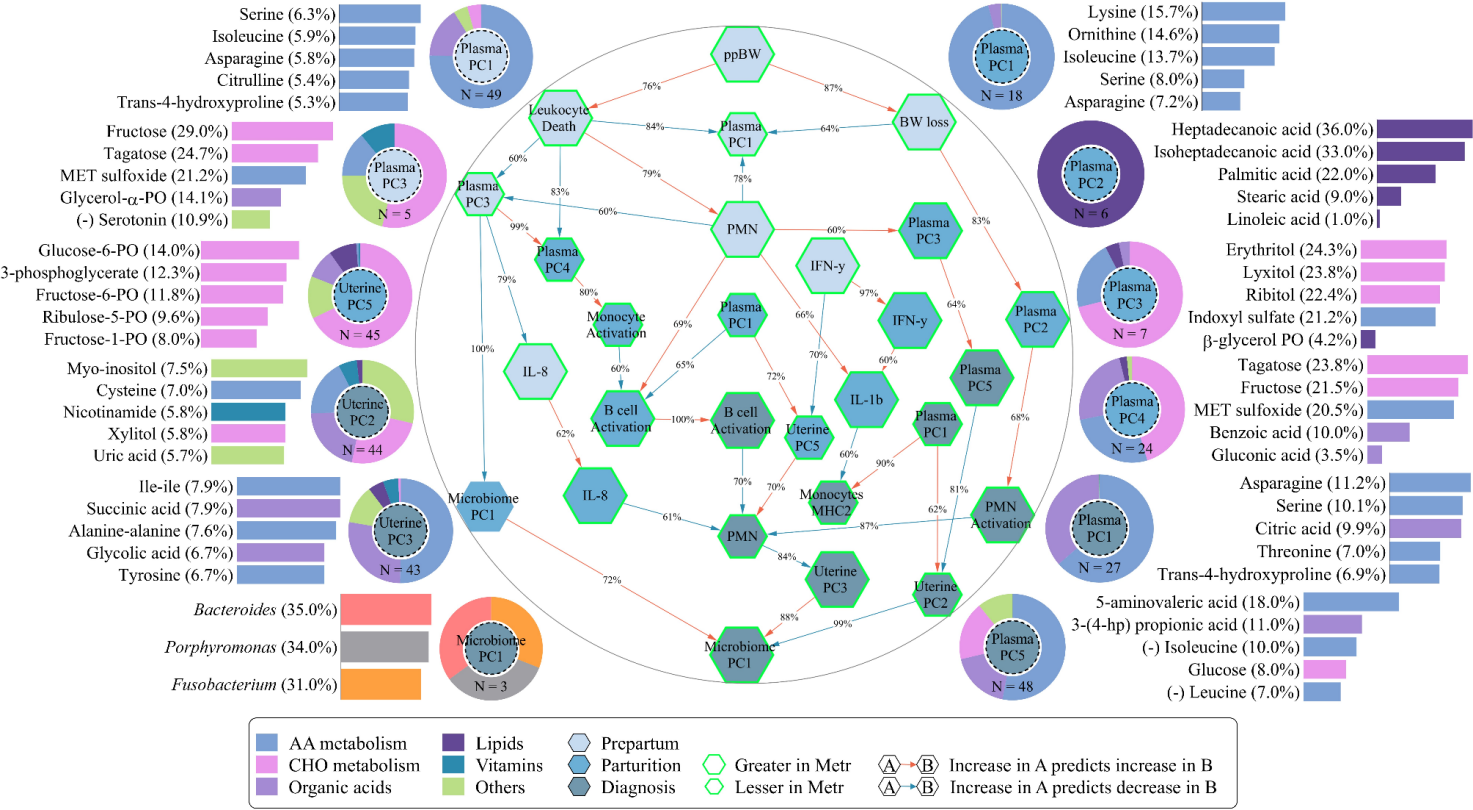
Bayesian network between immune variables, metabolome and microbiome latent variables, and metadata variables bootstrapped 1,000 times to estimate the uncertainty of the edge’s strength and the direction of the network. Hexagons represent nodes and arrows represent edges. Edges showing presence in at least 600 of the 1,000 models (60% strength) are shown. Average model estimates were extracted to determine if the parent node was positively or negatively predicting the child node. Positive estimates are represented by red edges and negative estimates are represented by blue edges. Edge labels represent the strength of each edge. Sparse principal component analyses were performed for plasma and uterine metabolome latent variable identification. Plasma principal components (PCs) indicate plasma latent variables. Uterine PCs indicate uterine latent variables. Principal component analyses were performed on uterine *Bacteroides*, *Fusobacterium*, and *Porphyromonas* for latent variables identification. Microbiome PCs indicate the latent variables composed of *Bacteroides*, *Fusobacterium*, and *Porphyromonas*. Information regarding each latent variable are shown surrounding the Bayesian network. Each pie chart and bar graph represent one latent variable. Colors represent the categories of metabolites, or different microbes, respectively. The center color on each pie chart represents the different timepoints. The “N =” on each pie chart represents the number of metabolites or microbes composing the respective latent variable. Bar graphs represent the top 5 most important metabolites explaining the variation of each latent variable. Percentages between parentheses represent the proportion of the variation in the respective component explained by the metabolite or microbe. Metabolites starting with “(-)” were negatively associated with their respective latent variable. Otherwise, metabolites were positively associated with the latent variable. Hexagons with green borders correspond to variables that differed between cows that developed metritis and cows that did not develop metritis. MET: methionine. PO: phosphate. Hp: hydroxy phenyl. ppBW: prepartum body weight. BW: body weight. PMN: polymorphonuclear cells. IFN-γ: interferon gamma. IL: interleukin. MHC2: major histocompatibility complex class 2. AA: amino acids. CHO: carbohydrates. Metr: metritis. Monocyte activation indicates an increase in cluster of differentiation (CD) 62 L. PMN activation indicates an increase in CD62L. B-cell activation indicates a decrease in CD62L

For plasma metabolome prepartum, PC1 and PC3 remained in the Bayesian network. Plasma prepartum PC1 explained 14.7% of the variation in the data (Supplemental Table S1) and was composed of 49 metabolites, of which 75.3% were amino acids (Supplemental Table S2; Supplemental Figure S1A). Plasma prepartum PC3 explained 4.8% of the variation in the data (Supplemental Table S1) and was composed of 5 metabolites, of which 53.8% were carbohydrates (Supplemental Table S2; Supplemental Figure S1C). Both plasma prepartum PC1 (*P* = 0.01) and PC3 (*P* < 0.01) were lesser in cows that developed metritis when compared with cows that did not develop metritis (Fig. 2; Supplemental Table S4).

For plasma metabolome at parturition, PC1, PC2, PC3, and PC4 remained in the Bayesian network. Plasma PC1 at parturition explained 11.0% of the variation in the data (Supplemental Table S1) and was composed of 18 metabolites, of which 95.9% were amino acids (Supplemental Table S2; Supplemental Figure S2A). Plasma PC2 at parturition explained 8.6% of the variation in the data (Supplemental Table S1) and was composed of 6 metabolites, of which 100.0% were lipids (Supplemental Table S2; Supplemental Figure S2B). Plasma PC3 at parturition explained 6.9% of the variation in the data (Supplemental Table S1) and was composed of 7 metabolites, of which 71.2% were carbohydrates (Supplemental Table S2; Supplemental Figure S2C). Plasma PC4 at parturition explained 4.6% of the variation in the data (Supplemental Table S1) and was composed of 24 metabolites, of which 45.3% were carbohydrates (Supplemental Table S2; Supplemental Figure S2D). Plasma PC1 (*P* = 0.08) and PC4 (*P* = 0.04) at parturition were lesser, and PC2 (*P* = 0.02) and PC3 (*P* = 0.02) at parturition were greater in cows that developed metritis when compared with cows that did not develop metritis (Fig. 2; Supplemental Table S4).

For plasma metabolome at diagnosis, PC1 and PC5 remained in the Bayesian network. Plasma PC1 at diagnosis explained 11.7% of the variation in the data (Supplemental Table S1) and was composed of 27 metabolites, of which 63.6% were amino acids (Supplemental Table S2; Supplemental Figure S3A). Plasma PC5 at diagnosis explained 3.9% of the variation in the data (Supplemental Table S1) and was composed of 48 metabolites, of which 53.1% were amino acids (Supplemental Table S2; Supplemental Figure S3E). Plasma PC1 at diagnosis (*P* < 0.01) was lesser and PC5 at diagnosis (*P* < 0.01) was greater in cows that developed metritis when compared with cows that did not develop metritis (Fig. 2; Supplemental Table S4).

For uterine metabolome at parturition, PC5 remained in the Bayesian network. Uterine PC5 at parturition explained 4.0% of the variation in the data (Supplemental Table S1) and was composed of 45 metabolites, of which 67.9% were carbohydrates (Supplemental Table S3; Supplemental Figure S4E). Uterine PC5 at parturition was lesser (*P* = 0.04) in cows that developed metritis when compared with cows that did not develop metritis (Supplemental Table S4).

For uterine metabolome at diagnosis, PC2 and PC3 remained in the Bayesian network. Uterine PC2 at diagnosis explained 15.5% of the variation in the data (Supplemental Table S1) and was composed of 44 metabolites, of which 28.6% were uncategorized metabolites (Supplemental Table S3; Supplemental Figure S5B). Uterine PC3 at diagnosis explained 7.5% of the variation in the data (Supplemental Table S1) and was composed of 43 metabolites, of which 50.2% were amino acids (Supplemental Table S3; Supplemental Figure S5C). Uterine metabolome PC2 at diagnosis (*P* < 0.01) was lesser and uterine PC3 at diagnosis (*P* < 0.01) was greater in cows that developed metritis when compared with cows that did not develop metritis (Fig. 2; Supplemental Table S4).

For uterine microbiome at parturition, PC1 was composed of 3 bacteria. By design, the 3 bacteria explained 100% of the variation in PC1. These bacteria were *Porphyromonas* (34.3%), *Bacteroides* (33.5%), and *Fusobacterium* (32.2%). For uterine microbiome at diagnosis, PC1 was composed of 3 bacteria. The 3 bacteria, which explained 100% of the variation in PC1, were *Bacteroides* (34.9%), *Porphyromonas* (34.5%), and *Fusobacterium* (30.6%).

### Bayesian network

A total of 55 edges connected 37 nodes with a strength greater or equal to 60% (Supplemental Table S5). Only 39 edges and 28 nodes remained in the final network (Fig. 2) because they were part of a pathway connected to Microbiome PC1 on the day of metritis diagnosis.

In the prepartum, ppBW and IFN-γ were not impacted by other nodes. Body weight change and circulating leukocyte viability were negatively impacted by ppBW. Polymorphonuclear cells were negatively impacted by circulating leukocyte viability. Plasma PC1, composed of 75.3% amino acids, was positively impacted by BWC and circulating leukocyte viability, and negatively impacted by PMN. Plasma PC3, composed of 53.8% carbohydrates, was positively impacted by circulating leukocyte viability and negatively impacted by PMN. Interleukin 8 was negatively impacted by plasma PC3.

At parturition, plasma PC1, composed of 95.9% amino acids, was not impacted by other nodes. Microbiome PC1, composed of *Bacteroides*, *Porphyromonas*, and *Fusobacterium*, was negatively impacted by prepartum plasma PC3. IL-8 was positively impacted by prepartum IL-8. Plasma PC4, composed of 45.3% carbohydrates, was positively impacted by prepartum circulating leukocyte viability and prepartum plasma PC3. Monocyte activation was positively impacted by plasma PC4. B-cell activation was negatively impacted by Monocyte activation and plasma PC1 and positively impacted by prepartum PMN. Uterine PC5, composed of 67.9% carbohydrates, was negatively impacted by prepartum IFN-γ and positively impacted by plasma PC1. Interferon γ was positively impacted by prepartum IFN-γ. Interleukin 1β was positively impacted by IFN-γ. Plasma PC2, composed of 100.0% lipids, was negatively impacted by BWC, and plasma PC3, composed of 71.2% carbohydrates, was positively impacted by prepartum PMN.

On the day of metritis diagnosis, plasma PC1, composed of 63.6% amino acids, was not impacted by other nodes. B-cell activation was positively impacted by parturition B-cell activation. Monocytes MHCII was positively impacted by plasma PC1 and negatively impacted by parturition IL-1β. Plasma PC5, composed of 53.1% amino acids, was positively impacted by parturition plasma PC3. Uterine PC2, composed of 28.6% uncategorized metabolites, was positively impacted by plasma PC1 and negatively impacted by plasma PC5. Polymorphonuclear cells activation was positively impacted by parturition plasma PC2. Polymorphonuclear cells were negatively impacted by B-cell activation, PMN activation, and parturition IL-8 and positively impacted by uterine PC5. Uterine PC3, composed of 50.2% amino acids, was negatively impacted by PMN. Microbiome PC1, composed of *Bacteroides*, *Porphyromonas*, and *Fusobacterium*, was negatively impacted by uterine PC2, and positively impacted by uterine PC3 and parturition microbiome PC1.

## Discussion

Herein, Bayesian networks were applied to infer potential causal relationships between the uterine microbiome, uterine and plasma metabolome, and systemic immune profiling to advance the understanding of metritis development in dairy cows. Bayesian network analysis is a powerful statistical method that can suggest potential causal relationships among variables, which can aid in the development of strong mechanistic hypotheses for further experimental testing [21].

According to the directionality network, the prepartum immune and metabolic status of cows were impacted by their ppBW. In dairy cows, body weight is strongly correlated with body condition score (BCS), a measure of subcutaneous adiposity [11]. The greater ppBW in cows that developed metritis negatively impacted the prepartum circulating leukocyte viability and the BWC leading to heavier cows experiencing more leukocyte death and greater BW loss. The greater level of fatty acid accumulation in adipocytes from fatter animals activates NADPH oxidase, increasing the production of reactive oxygen species (ROS) and their release to circulation [24]. Elevated systemic ROS levels cause cell death [24]. Furthermore, cows with greater BCS have more circulating leptin, which has been proposed as a reason why these over-conditioned prepartum cows experience a more pronounced reduction in feed intake and negative nutrient balance prior to parturition, leading to greater BW loss [11]. The greater leukocyte death observed in cows that developed metritis led to an increase in prepartum PMN. Following cell death, intracellular components are released to circulation triggering immune cell recruitment [25]. It is possible that PMN were released from the bone marrow or spleen as a response to leukocyte death-associated oxidative stress [26]. Greater leukocyte death and PMN led to a reduction in prepartum plasma PC3, which was composed of fructose, tagatose, methionine sulfoxide, glycerol-α-phosphate, and serotonin. All these metabolites except for serotonin had positive loadings (i.e. positive correlations), whereas serotonin had a negative loading, meaning that the observed reduction in prepartum plasma PC3 in cows that developed metritis indicates a reduction in all of these metabolites but an increase in serotonin. Fructose is a sugar that can enter the glycolytic pathway at various points [27], while glycerol-α-phosphate is an intermediate in glycolysis. The observed decrease of these metabolites may reflect an overall usage of these glycolytic substrates by PMN, which upon activation shift their metabolism from oxidative phosphorylation to glycolysis [28]. Under conditions of elevated ROS and cell death, methionine residues in proteins are oxidized to methionine sulfoxide [29]. In an attempt to reverse the oxidative damage, methionine sulfoxide reductases catalyze the reduction of methionine sulfoxide back to methionine, thereby, reducing methionine sulfoxide concentrations [29]. The observed decrease in methionine sulfoxide levels in cows experiencing greater leukocyte death may indicate a response to a greater oxidative challenge. Although most of the circulating serotonin is synthetized by the enterochromaffin cells of the gastrointestinal tract, leukocytes are able to synthesize and store serotonin [30]. It is possible that following leukocyte death, intracellular serotonin is released; thus, increasing circulating serotonin levels. Serotonin enhances the production of IL-8 by dendritic cells [31], which may help explain why plasma PC3 led to an increase in prepartum IL-8.

According to the directionality network, the immune and metabolic status of cows at parturition were impacted by changes observed in the prepartum. The greater BW loss in cows that developed metritis led to an increase in plasma PC2 at parturition. Plasma PC2 was composed of fatty acids, indicating that heavier cows were losing more weight, consequently mobilizing more adipose tissue. Furthermore, the greater prepartum leukocyte death and the reduction in prepartum plasma PC3 led to a decrease in plasma PC4 at parturition. Similar to prepartum plasma PC3, plasma PC4 at parturition was mostly composed of tagatose, fructose, and methionine sulfoxide, which indicates that the response to oxidative stress observed in the prepartum was maintained at parturition. The decrease in parturition plasma PC4 led to a reduction in monocyte activation at parturition. It is possible that the reduction in glycolytic substrates hindered monocyte activation. Contrarily to neutrophils, monocytes lack glycogen storages; thus, they depend entirely on extracellular carbohydrates for activation [32]; making them more sensible to low carbohydrate levels. The decrease in monocyte activation, the decrease in parturition plasma PC1, composed of amino acids, and the increase in prepartum PMN, which was impacted by prepartum leukocyte death, led to an increase in B-cell activation at parturition. Although not included in the Bayesian network analysis presented herein because of the high collinearity with B-cell activation at parturition, B-cell activation prepartum was directly impacted by prepartum leukocyte death (data not shown), indicating that B-cell activation is part of the immune response to leukocyte death [25, 33]. The decrease in prepartum plasma PC3 led to an increase in parturition microbiome PC1, composed of *Bacteroides*,* Porphyromonas*, and *Fusobacterium*. In humans, bacteria present in the gut sense serotonin levels, which impact gene expression related to biofilm formation, adhesion, motility, and virulence [34]. For example, serotonin impacts quorum sensing pathways increasing virulence of *Pseudomonas aeruginosa*, leading to *Pseudomonas aeruginosa* overgrowth [35]. At parturition, dairy cows have degenerative vascular changes in uterine small blood vessels [16], which has been proposed as a mechanism by which blood components leak into the uterine lumen [15]. It is possible that circulating serotonin seeps into the uterus and affects the uterine microbiome favoring opportunistic pathogenic bacteria overgrowth. It is worth noting that, on average, uterine samples were collected approximately 12 h after parturition, which allowed for bacterial proliferation [36]. In fact, we previously observed that uterine serotonin was greater in cows with metritis than in cows without metritis, and that was highly correlated with uterine pathogenic bacteria [6].

**Fig. 3:**
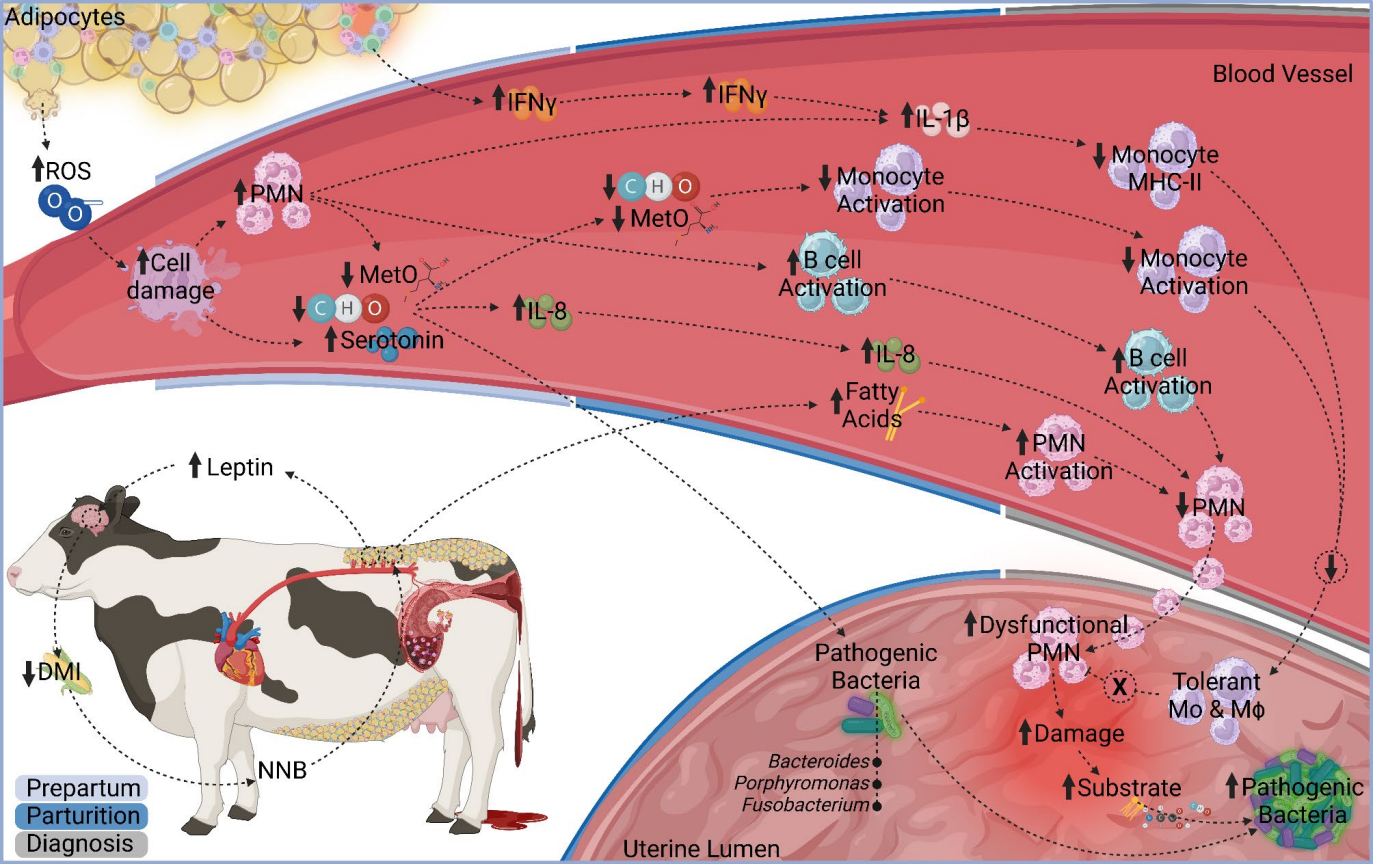
Illustration of our working hypothesis of the interactions between prepartum body weight, body weight loss, systemic immune profiling, and uterine and plasma metabolome around parturition and how they may affect uterine opportunistic pathogens growth leading to metritis development. Down arrows indicate reduction, and up arrows indicate increase in cows with metritis. ROS: reactive oxygen species. MetO: methionine sulfoxide. CHO: carbohydrates. IL: interleukin. PMN: polymorphonuclear cells. IFN: interferon. MHC: major histocompatibility complex. DMI: dry matter intake. NNB: negative nutrient balance. Mo: monocytes. Mϕ: macrophages. Figure created with Biorender

According to the directionality network, the immune and metabolic status of cows at the day of metritis diagnosis were impacted by changes observed in the prepartum and at parturition. The greater parturition plasma PC2, composed of fatty acids, led to a greater PMN activation at the day of metritis diagnosis in cows with metritis. Treating bovine neutrophils with long chain fatty acids increased their adhesion and chemotaxis [37], indicating a possible direct effect of greater circulating fatty acids on PMN activation. The observed greater B-cell activation at parturition was maintained at metritis diagnosis, and together with greater PMN activation at the day of metritis diagnosis, greater parturition IL-8, and lesser parturition uterine PC5, mostly composed of carbohydrates, led to a reduction in circulating PMN at metritis diagnosis. Greater levels of IL-8, which is the main PMN chemokine [38], and greater expression of CD62L, which is an adhesion molecule on PMN [10], likely promoted PMN migration to the uterus, leading to a reduction in circulating PMN. In fact, PMN are more abundant in the endometrium of cows with metritis than in the endometrium of cows without metritis [39]. Despite the greater PMN migration to the uterus in cows with metritis, circulating PMN from cows with metritis have lesser intracellular killing capacity when compared with cows without metritis [40]. It is possible that these dysfunctional PMN arriving in the uterus are less effective at eliminating pathogenic bacteria, resulting in a persistent chemo attractive signal for additional PMN recruitment. The lesser circulating PMN in cows that developed metritis led to an increase in uterine PC3 on the day of metritis diagnosis in cows with metritis, which consequently led to an increase in *Bacteroides*, *Porphyromonas*, and *Fusobacterium* at diagnosis. Uterine PC3 at diagnosis was mostly composed of amino and organic acids. Upon arrival to tissues, activated PMN release proteolytic enzymes [41], leading to endometrial damage [39]. Tissue damage triggers the breakdown of proteins, releasing dipeptides, such as ile-ile and alanine-alanine, and amino acids such as tyrosine and phenylalanine [42]. Furthermore, cellular stress and metabolic alterations during tissue injury leads to an increase in several other metabolites, including organic acids such as succinic and fumaric acids [43]. Greater tissue damage creates an environment that may promote opportunistic pathogenic bacterial proliferation by increasing substrate availability [44], which might explain how the greater levels of uterine PC3 in cows with metritis positively impacted the observed increase in *Bacteroides*, *Porphyromonas*, and *Fusobacterium*. The greater parturition IL-1β together with the lesser diagnosis plasma PC1, composed of amino and organic acids, led to a reduction in monocytes MHCII expression at diagnosis. The lesser circulating amino acids may impair monocyte antigen presentation capacity. It is also possible that these cells were experiencing immune tolerance. The greater parturition IL-1β was positively impacted by parturition IFN-γ, which was positively impacted by prepartum IFN-γ. Prepartum IFN-γ was not influenced by any node. The origin of the greater prepartum IFN-γ in cows that developed metritis is, therefore, not clear. In obese non-ruminants, T-cells surrounding necrotic adipocytes produce high levels of IFN-γ [45]; thus, we hypothesize that the greater prepartum IFN-γ in cows that developed metritis may originate from adipocyte-associated T-cells. Nonetheless, the persistent systemic inflammation observed since the prepartum led to a reduction in monocyte antigen presentation capacity. In a previous study, we hypothesized that the reduction in monocyte antigen presentation capacity observed postpartum in cows with metritis could be due to immune tolerance [10]. In fact, prolonged inflammatory responses are known for leading to immune tolerance [46], particularly reducing monocytes and macrophages activation [47] but not PMN activation [48]. It is noteworthy that we did not detect any influence of the reduction in monocyte antigen presentation capacity on the abundance of opportunistic pathogenic bacteria; however, it is possible that if circulatory monocytes are experiencing immune tolerance, uterine monocytes and macrophages could also be experiencing tolerance. Monocytes and macrophages play a key role in the resolution of inflammation [49]; therefore, their impaired response could hinder the resolution of inflammation, potentially leading to greater tissue damage by PMN. Our working hypothesis is illustrated in Fig. 3.

The main limitation of our study is the absence of BCS measurements, which led us to rely on pre- and post-partum BW and their difference as indicators of fat mobilization and energy balance. Therefore, future studies should consider using more reliable measures of energy status such as BCS, back-fat thickness, energy intake, and energy expenditure. Furthermore, the use of 16 S rRNA sequencing instead of whole metagenomic sequencing for microbial identification precluded us from investigating the association between specific microbial species and immune responses and specific metabolites in our Bayesian network analysis. Lastly, the use of a viability marker prior to gating the different leukocyte populations limited our ability to determine whether specific leukocytes were responsible for the observed differences in leukocyte death between cows with and without metritis. However, gating leukocytes without first applying a viability marker would have likely produced inaccurate results due to non-specific antibody binding by dead cells. Of note as well is the fact that our Live/Dead™ staining method based on esterase activity and plasma membrane integrity is limited in its ability to distinguish between live and apoptotic cells; therefore, apoptotic cells may be classified as live cells.

## Conclusions

This observational study provides insights into the complex relationship between ppBW, BW loss, systemic immune profiling, and uterine and plasma metabolome around parturition, and how they may promote uterine opportunistic pathogens growth leading to metritis development. Altogether, directionality network analysis indicates that greater prepartum BW led to greater BW loss and prepartum systemic cellular death, which led to an increase in systemic inflammation and immune activation. The aforementioned changes led to an increase in PMN extravasation, which led to an increase in uterine metabolomic changes associated with tissue damage. These changes led to an increase in *Bacteroides*, *Porphyromonas* and *Fusobacterium* in cows that developed metritis, indicating that excessive tissue damage may promote opportunistic pathogenic bacterial growth. Experimental research needs to be conducted to validate the mechanistic hypotheses generated herein. In conclusion, the directionality network showed that prepartum BW led to immune dysregulation and plasma and uterine metabolomic changes leading to opportunistic pathogens overgrowth in cows that developed metritis.

## Electronic supplementary material

Below is the link to the electronic supplementary material.

Supplementary Material 1

Supplementary Material 2

Supplementary Material 3

Supplementary Material 4

Supplementary Material 5

Supplementary Material 6

Supplementary Material 7

## Data Availability

The raw sequence data used in the current study were generated during our previous study [6] and are available in the NCBI repository under BioProject OR883023 (www.ncbi.nlm.nih.gov/nuccore/OR883023) - OR883397 (www.ncbi.nlm.nih.gov/nuccore/OR883397). The metabolomics dataset used in the current study was generated during our two previous studies [6, 13] and are available in the NIH Common Fund’s National Metabolomics Data Repository website, the Metabolomics Workbench repository [50]. Uterine metabolomics dataset [6] can be found under Study ID ST002994, http://dx.doi.org/10.21228/M8S425. Plasma metabolomics dataset [13] can be found under Study ID ST002556; http://dx.doi.org/10.21228/M8PF0K. The Metabolomics Workbench is supported by NIH grant U2C-DK119886 and OT2-OD030544 grants.

## Abbreviations

BW: Body weight
ppBW: Prepartum body weight
BWC: Daily body weight change as a percentage of calving body weight
PCA: Principal component analysis
sPCA: Sparse Principal component analysis
BIC: Bayesian information criterion
PC: Principal component
BCS: Body condition score
ROS: Reactive oxygen species
