# Supplementary material for: Multi-omics integration and immune profiling identify possible causal networks leading to uterine microbiome dysbiosis in dairy cows that develop metritis
Source: Anim Microbiome. 2025 Jan 9;7:4. doi: 10.1186/s42523-024-00366-9 (PMC11716391; doi:10.1186/s42523-024-00366-9)
Supplement: Supplementary file 6 — Supplementary Material 6 [file 42523_2024_366_MOESM6_ESM.pdf]

SUPPLEMENTAL FIGURES

Multi-omics integration and immune profiling identify possible causal networks leading to uterine microbiome dysbiosis in dairy cows that develop metritis

S. Casaro[1], J. G. Prim[2], T. D. Gonzalez[1], F. Cunha[1], A. C. M. Silva[3], H. Yu[3], R. S. Bisinotto[1], R. C. Chebel[1], J. E. P. Santos[3,4], C. D. Nelson[3], S. J. Jeon[5], R. C. Bicalho[6], J. P. Driver[7], K. N. Galvão[1,4]*

[1]Department of Large Animal Clinical Sciences, University of Florida, Gainesville, FL, 32610

[2]Department of Clinical Sciences, Auburn University, Auburn, AL, 36849

[3]Department of Animal Sciences, University of Florida, Gainesville, FL, 32610

[4]D. H. Barron Reproductive and Perinatal Biology Research Program, University of Florida, Gainesville, FL, 32610

[5]Department of Veterinary Biomedical Sciences, Long Island University, Brookville, NY, 11548

[6]FERA Diagnostics and Biologicals, College Station, TX, 77845

[7]Division of Animals Sciences, University of Missouri, Columbia, MO, 65211

*Corresponding author: Klibs N. Galvão: galvaok@ufl.edu

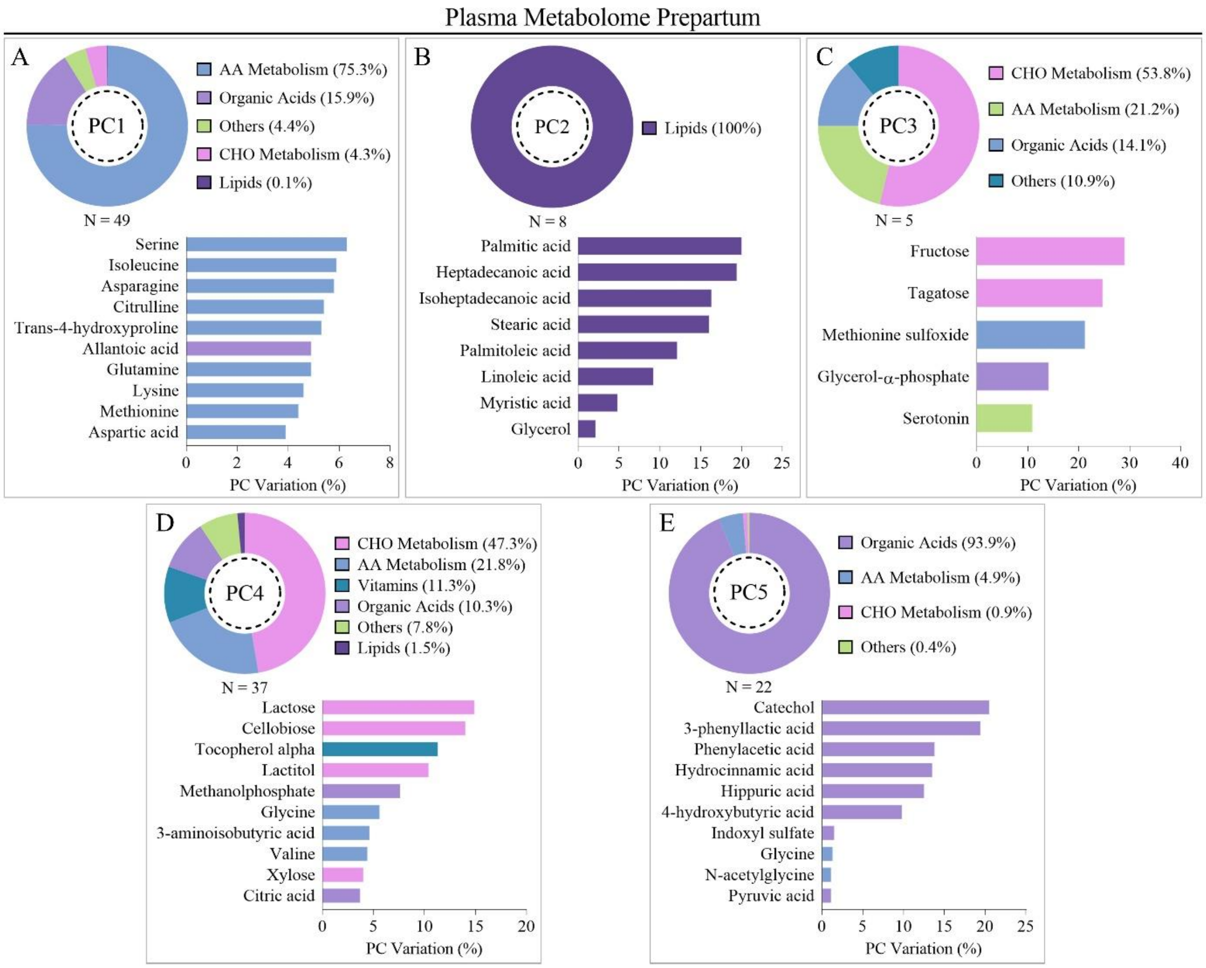

Supplemental Figure 1. Principal components (PC) generated by sparce PC analysis for the plasma metabolome in the prepartum. Panels A, B, C, D, and E show PC1, 2, 3, 4, and 5, respectively. AA, amino acids; CHO, carbohydrates. Figure created using GraphPad Prism version 8.4.3 for Windows, GraphPad Software, San Diego, California, USA.

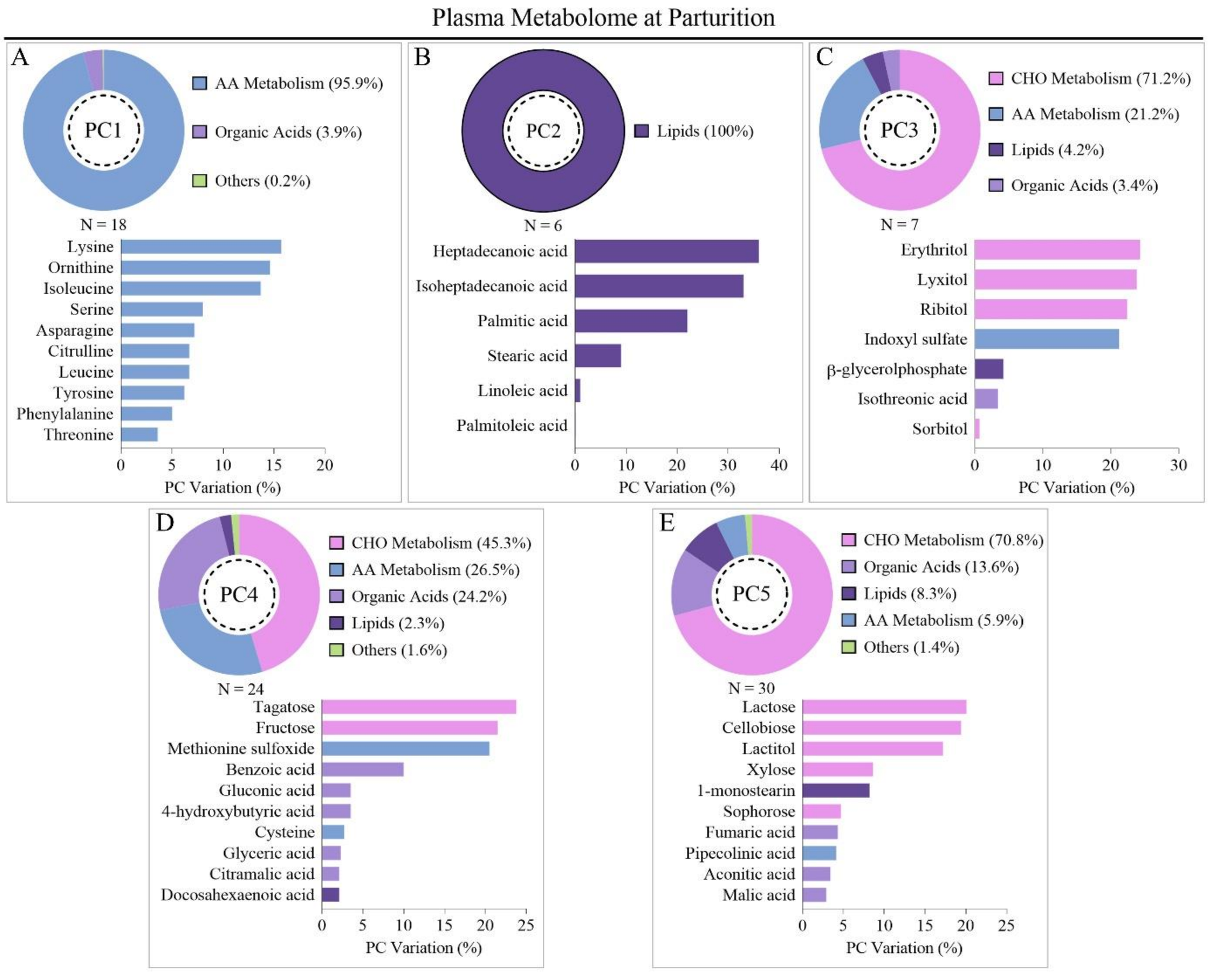

Supplemental Figure 2. Principal components (PC) generated by sparce PC analysis for the plasma metabolome at parturition. Panels A, B, C, D, and E show PC1, 2, 3, 4, and 5, respectively. AA, amino acids; CHO, carbohydrates. Figure created using GraphPad Prism version 8.4.3 for Windows, GraphPad Software, San Diego, California, USA.

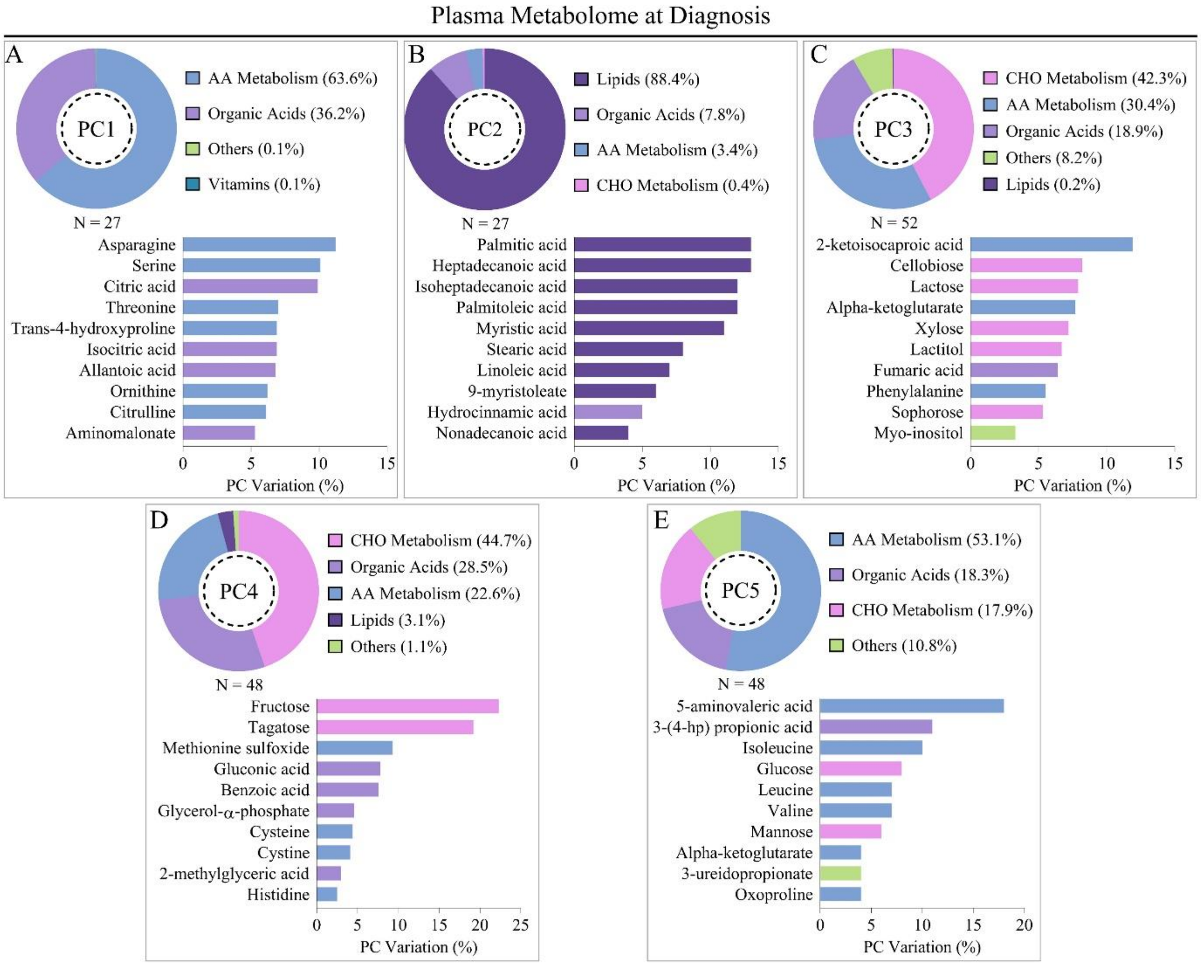

Supplemental Figure 3 Principal components (PC) generated by sparce PC analysis for the plasma metabolome on the day of metritis diagnosis. Panels A, B, C, D, and E show PC1, 2, 3, 4, and 5, respectively. AA, amino acids; CHO, carbohydrates. Figure created using GraphPad Prism version 8.4.3 for Windows, GraphPad Software, San Diego, California, USA.

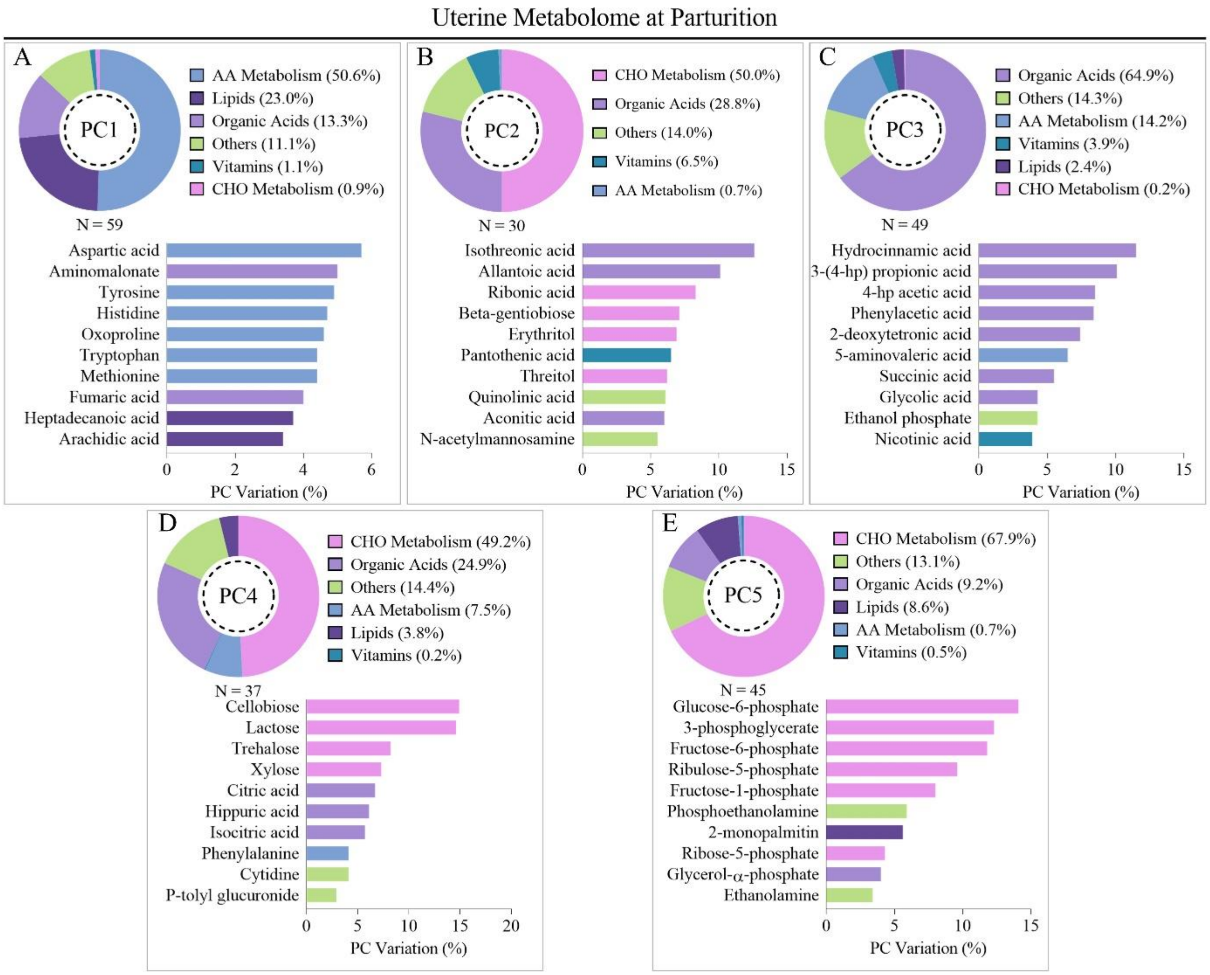

Supplemental Figure 4 Principal components (PC) generated by sparce PC analysis for the uterine metabolome at parturition. Panels A, B, C, D, and E show PC1, 2, 3, 4, and 5, respectively. AA, amino acids; CHO, carbohydrates. Figure created using GraphPad Prism version 8.4.3 for Windows, GraphPad Software, San Diego, California, USA.

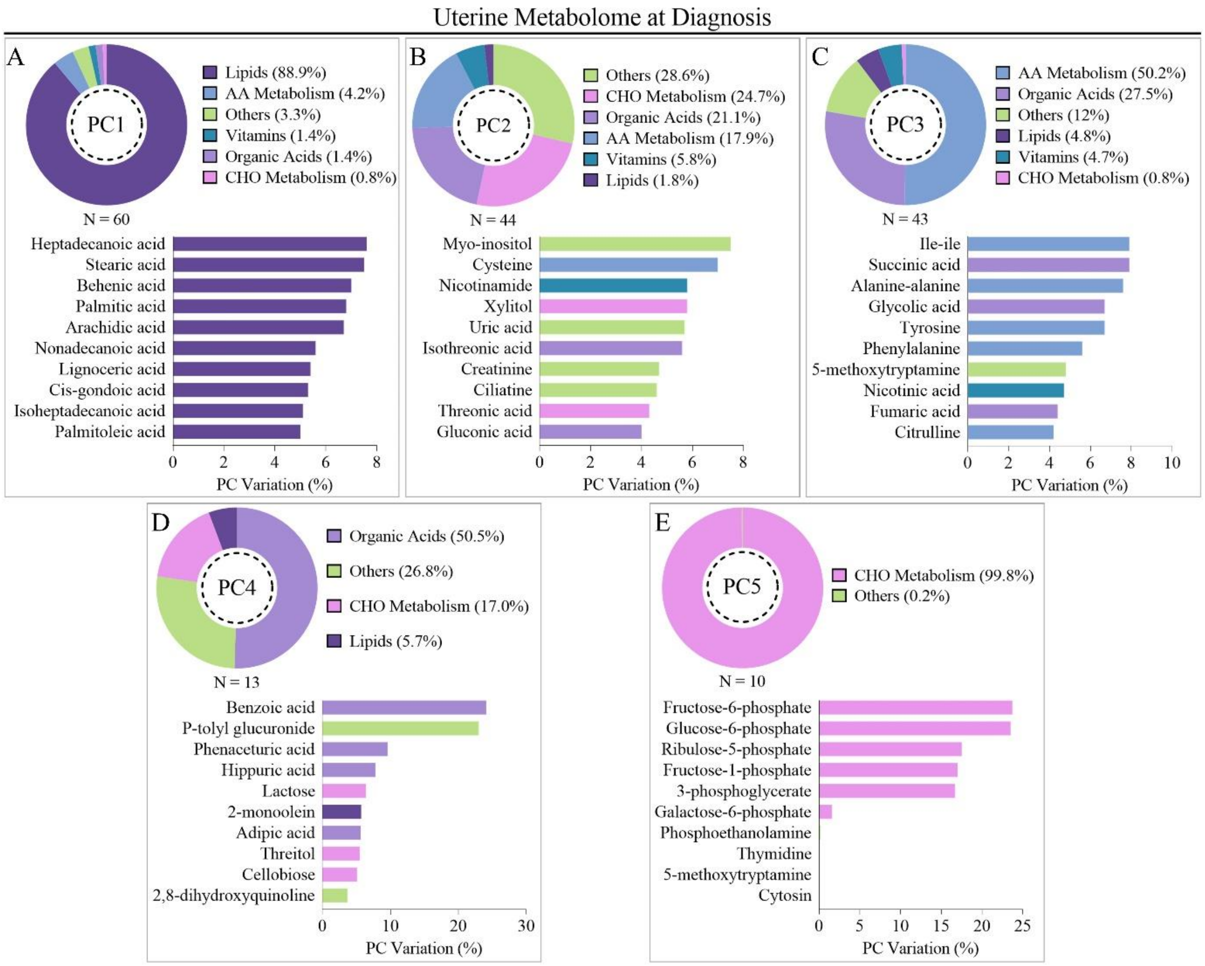

Supplemental Figure 5 Principal components (PC) generated by sparce PC analysis for the uterine metabolome on the day of metritis diagnosis. Panels A, B, C, D, and E show PC1, 2, 3, 4, and 5, respectively. AA, amino acids; CHO, carbohydrates. Figure created using GraphPad Prism version 8.4.3 for Windows, GraphPad Software, San Diego, California, USA.
