# Supplementary material for: Multi-omics integration and immune profiling identify possible causal networks leading to uterine microbiome dysbiosis in dairy cows that develop metritis
Source: Anim Microbiome. 2025 Jan 9;7:4. doi: 10.1186/s42523-024-00366-9 (PMC11716391; doi:10.1186/s42523-024-00366-9)
Supplement: Supplementary file 7 — Supplementary Material 7 [file 42523_2024_366_MOESM7_ESM.pdf]

MATERIALS AND METHODS

Multi-omics integration and immune profiling identify possible causal networks leading to uterine microbiome dysbiosis in dairy cows that develop metritis

S. Casaro[1], J. G. Prim[2], T. D. Gonzalez[1], F. Cunha[1], A. C. M. Silva[3], H. Yu[3], R. S. Bisinotto[1], R. C. Chebel[1], J. E. P. Santos[3,4], C. D. Nelson[3], S. J. Jeon[5], R. C. Bicalho[6], J. P. Driver[7], K. N. Galvão[1,4]*

[1]Department of Large Animal Clinical Sciences, University of Florida, Gainesville, FL, 32610

[2]Department of Clinical Sciences, Auburn University, Auburn, AL, 36849

[3]Department of Animal Sciences, University of Florida, Gainesville, FL, 32610

[4]D. H. Barron Reproductive and Perinatal Biology Research Program, University of Florida, Gainesville, FL, 32610

[5]Department of Veterinary Biomedical Sciences, Long Island University, Brookville, NY, 11548

[6]FERA Diagnostics and Biologicals, College Station, TX, 77845

[7]Division of Animals Sciences, University of Missouri, Columbia, MO, 65211

*Corresponding author: Klibs N. Galvão: galvaok@ufl.edu

Cows, Housing, and Feeding

The study included 71 primigravid and 57 multigravid cows, beginning at 240 days of gestation and concluding at 13 ± 1 days after parturition. Throughout the study period, all cows were housed in free-stall barns with sand-bedded stalls pre- and postpartum. Prepartum, multigravid cows were fed a TMR formulated to meet or exceed the nutrient requirements for dry Holstein cows weighing 680 kg [1] twice daily. Postpartum, multiparous cows were fed a TMR formulated to meet or exceed the nutrient requirements for lactating Holstein cows weighing 680 kg and producing 45 kg of 3.5% fat-corrected milk [1] twice daily. Nulliparous cows were housed in a free-stall barn with individual feeding gates (Calan Broadbent Feeding System, American Calan Inc., Northwood, NH) since 241 d of gestation and were fed a TMR formulated to meet or exceed the nutrient requirements for dry Holstein heifers weighing 650 kg once daily. After parturition, primiparous cows were moved to a postpartum pen also equipped with individual feeding gates and each cow was assigned to an individual gate until 100 DRP. Primiparous cows were fed a TMR formulated to meet or exceed the nutrient requirements for lactating Holstein cows weighing 600 kg and producing 35 kg of 3.5% fat-corrected milk [1] twice daily. Nulliparous animals were part of an experiment where they were assigned to a control, which was offered 100 g/cow/d ground corn as a top-dress to the total mixed diet, and to a treatment, which consisted of top-dress addition of calcidiol (ROVIMIX Hy-D 1.25%. 12.5 mg/g calcidiol; DSM Nutritional Products, Parsippany, NJ) at a concentration of 10 mg/kg ground corn from 248 d of gestation until 100 DRP.

Postpartum, all cows were milked two times a day at 0600 and 1800 h. The yearly rolling herd average milk yield was approximately 11,000 kg.

Exclusion Criteria

Cows that developed mastitis, digestive problems, or respiratory disease in the first 35 days after calving, cows that received antimicrobial treatment before metritis diagnosis, and cows diagnosed with metritis after 10 days after calving were excluded from the study. Cows that developed metritis after day 10 were excluded because sampling was only performed up to day 10. Exclusion of cows diagnosed with metritis after day 10 ascertained that these cows would not be misclassified as healthy cows in our analysis. Cows with missing values in any of the measured responses were excluded. A total of 15 cows were excluded. Four cows were excluded because they were treated with antimicrobials before metritis diagnosis. Three cows were excluded because of death. One cow was excluded because of uterine torsion and one cow was excluded because of peritonitis. Four cows were excluded because they were diagnosed with metritis at 13 ± 1 days after calving. Two cows were excluded because of missing data points in some immune cell populations. After exclusions, there were 113 cows available; 50 that did and 63 that did not develop metritis.

Uterine Discharge Evaluation

Uterine discharge was evaluated using a Metricheck device (Metricheck, Simcro, New Zealand) at 3 ± 1, 7 ± 1, 10 ± 1 and 13 ± 1 days after parturition using a 5-point scale as previously described [2]: 1 = not fetid normal lochia, viscous, clear, red, or brown; 2 = cloudy, pink, red, or brown mucoid discharge with flecks of pus; 3 = not fetid, pink red or brown mucopurulent discharge with < 50% pus; 4 = not fetid, pink, red or brown purulent discharge with ≥ 50% pus; 5 = fetid red-brownish, watery discharge.

Case Definition and Control Selection

Cows with a uterine discharge score of 5 in at least one examination were classified as having metritis and cows with a discharge score ≤ 4 were classified as not having metritis. Cows

diagnosed with metritis up to day 10 were considered cases for this study. There were 52 metritis cases, and 10, 23, and 19 cows were diagnosed with metritis on day 3, 7, and 10, respectively. Two cows were excluded because of several missing values for Bayesian networks on the day of metritis diagnosis. Control cows (n = 50) that did not develop metritis were matched with 50 cows that developed metritis, hence, 100 cows were used for bioinformatic and statistical analyses. An attempt was made to pair cows that did and did not develop metritis according to the day of metritis diagnosis, although it was not always possible. Of the control cows, 10, 30, and 10 cows were sampled on day 3, 7, and 10, respectively. Of these 50 cows, 14, 16, 13, and 7 cows had a uterine discharge score of 1, 2, 3, and 4, respectively.

Blood Sample Collection

All cows had blood collected prepartum (14±6 d before parturition), at parturition (first 24 hours after parturition), and at the day of metritis diagnosis (7±2 d after parturition). Blood was sampled from the jugular vein using a 20-gauge x 2.54-cm needle and 10-mL evacuated tube containing lithium heparin (Vacutainer, Becton, Dickinson and Company, Franklin Lakes, NJ, USA). To avoid contamination, a 10 cm^2 area over the jugular vein was prepared by removing the hair using clippers and scrubbing with alcohol-soaked gauze pads. After collection, two blood tubes were placed on ice and transported to the laboratory within 2 hours. Once in the laboratory, two 100-µL aliquots of blood per cow were immediately extracted from one blood tube and used for flow cytometric analysis. One blood tube was centrifuged at 4000 g, 4 °C, for 10 min, and the plasma was stored in two aliquots at -80 °C for further characterization of the plasma metabolome and cytokine profile.

Blood Flow Cytometry Analysis

Blood Immune cell populations were characterized using a Thermofisher Attune Nxt Flow Cytometer as previously described [3, 4]. Briefly, once red blood cells were lysed, cells

were incubated for 20 min at room temperature with a LIVE/DEAD™ Near-IR dead cell stain kit (Thermo Fisher Scientific, Waltham, MA) to stain dead cells. Afterward, the cells were washed with PBS and incubated for 10 min at 4°C with 10 µg of polyclonal rat IgG (I4131, Sigma-Aldrich, Saint Louis, MO) to block non-specific Ab binding, and cells were then stained and incubated for 30 min at 4 °C with one of two antibody panels. Panel 1 contained Ab against CD4, CD8α, TCR-δ chain, and CD62L and panel 2 contained Ab against CD21, MHCII, CD172α, CD14, CD11b, and CD62L. Gating strategy used can be found in our previous study [4]. Briefly, on the first panel, once live and single cells were gated, myeloid cells were identified as CD172α^{+}. Monocytes were identified as CD172α^{+}/CD14^{+} and because polymorphonuclear cells (PMN) did not express CD14, they were identified using side scatter (SSC) as CD172α^{+}/CD14^{-}/SSC^{high}. The proportion of both monocytes and PMN was calculated from the live/singlet cells. Lymphocytes were gated from the live/singlet cells using forward scatter (FSC) and SSC and within the lymphocytes, B-cells were identified as MHCII^{+}/CD21^{+}. The proportion and median fluorescence intensity (MFI) of CD11b and CD62L were used as markers of cell activation on the previously gated populations. Monocytes and PMN with greater CD11b MFI were considered as activated [5, 6]. Monocytes and PMN with greater CD62L MFI were considered as activated [7, 8]. The proportion and MFI of MHCII were also recorded on the monocytes. Monocytes with greater MHCII^{+} proportion or MFI were considered as being more capable to present antigenic peptides [9]. B-cells with lesser CD62L^{+} proportion or MFI and greater CD11b^{+} proportion or MFI were considered as activated. [10, 11].

On the second panel, once live and single cells were gated, lymphocytes were identified using FSC and SSC parameters. Within the lymphocyte gated population, CD4^{+} T-cells were gated using CD4, CD8^{+} T-cells were gated using CD8α, and γδ T-cells were gated using γδTCR.

The proportion of parent and MFI were recorded for CD4, CD8, and γδ T-cells. The proportion and MFI of CD62L were used as markers of cell activation on the previously gated populations. Lymphocytes lacking CD62L or with lesser MFI were considered as activated [12].

Data were analyzed using FlowJo software (Version 10.0.7, Treestar, Palo Alto, CA). Once data tables were extracted from FlowJo, the average between the two panels live cells, singlets, and lymphocytes were calculated and used.

The flow cytometer settings were configured to collect a volume of 200 µL of sample or a maximum of 100,000 cells, whichever was reached first. The volume of fluid utilized to capture the 100,000 cells was recorded and whenever the total number of cells was less than 100.000, the total number of cells within the 200 µL sample was recorded.

Plasma Cytokine Analysis

One frozen plasma aliquot was submitted to the University of Florida's Proteomics & Mass Spectrometry core (RRID:SCR_019151) of the Interdisciplinary Center for Biotechnology Research for multiplex analysis. A MILLIPLEX Bovine Cytokine/Chemokine 08-plex kit (BCYT1-33 K-08; EMD Millipore Corporation, Billerica, MA, USA) utilizing antibodies to bovine IFN-γ, IL-1α, IL-1β, IL-4, IL-6, IL-8, IL-10, and TNF-α was used to screen one of the stored plasma aliquots. The assay was performed according to the manufacturer's instructions by blinded technicians. Concentrations of markers were measured on the Luminex FlexMap 3D instrument (Luminex Corporation, Austin, TX, USA) in pg/mL. Quality control values ranged between 6.0 and 28.8, 12.6 and 70.2, 104.5 and 543.9, 618.0 and 3334.6, 119.8 and 619.2, 129.1 and 531.8, 40.4 and 209.5, 426.7 and 2594.6 for IFN-γ, IL-1α, IL-1β, IL-4, IL-6, IL-8, IL-10, and TNF-α, respectively, which were within the ranges indicated by the manufacturer. Inter-assay coefficients of variation were 3%, 3%, 1%, 7%, 2%, 3%, 5%, and 4% for IFN-γ, IL-1α, IL-1β, IL-4, IL-6, IL-8, IL-10, and TNF-α, respectively. Intra-assay coefficients of variation were

11.5%, 13.3%, 12.5%, 9.5%, 6.9%, 3.6%, 5.1%, and 14.0% for IFN-γ, IL-1α, IL-1β, IL-4, IL-6, IL-8, IL-10, and TNF-α, respectively.

Plasma Metabolome Analysis

The second frozen plasma aliquot was submitted to the University of California's West Coast Metabolomics Center in Davis, CA for metabolome analysis. Samples were analyzed by blinded technicians using untargeted GC-TOF-MS in a single batch as previously described [13, 14]. The carrier selected was helium gas, and a column comprised of 95% dimethyl/5diphenyl polysiloxanesne was used. Column flow-rate was set at 1 mL/min, and initial oven temperature was set at 50°C followed by a 20°C increase per min up to a final temperature of 330°C, which was held constant for a period of 5 min. Injection temperature was set to begin at 50°C followed by a 12°C increase per second up to 250°C. Retention of primary metabolites was evaluated using default settings from ChromaTOF v. 2.32 and quantification was reported as peak height. Each metabolite was identified based on its mass and charge relationship. Metabolites were annotated using PubChem, KEGG, and HMDB. Of the 1,014 detected metabolites, a total of 166 metabolites were annotated, and 848 were unknown. The metabolome dataset and metadata are available at the NIH Common Fund's National Metabolomics Data Repository website, the Metabolomics Workbench [15], under Study ID ST002556; DOI: 10.21228/M8PF0K.

Uterine Fluid Collection

All cows had uterine fluid collected at parturition and at diagnosis of metritis. Briefly, the cow's cervix was stabilized by rectal palpation, the vulva was rinsed with alcohol 70% (vol/vol) and dried with paper towels. Subsequently, a single-use plastic round-tip pipette (UterFlush pipettes, Van Beek) was introduced into the vagina at a 45° angle and manipulated through the cervix. A total of 50 mL of sterile saline solution (0.9% sodium chloride irrigation, Baxter) was infused into the uterine lumen using a 60-mL syringe (Covidien) attached to the end of the

pipette. Uterine contents were homogenized, retrieved into the same 60-mL syringe, and transferred to a sterile 15-mL conical tube (VWR). After collection, tubes were placed on ice and transported to the laboratory within 2 hours. Once in the laboratory, uterine fluid samples were aliquoted into 2-mL microcentrifuge tubes (Eppendorf) and stored at -80 ^{o}C until essayed.

Uterine Microbiome Analysis

One frozen uterine fluid aliquot was submitted to FERA Diagnostics and Biologicals Corporate in College Station, Texas for microbiome analysis. Samples were analyzed by technicians blinded to study groups. DNA extraction was performed using a Mag-Bind Universal Pathogen 96 Kit (Omega Bio-Tek, Norcross, GA) in accordance with manufacturer instructions. The 16S rRNA gene was amplified by PCR. Amplification of the V4 hypervariable region of the bacterial/archaeal 16S rRNA gene was performed as previously described [16] using the Illumina MiSeq platform (Illumina Inc.). Description of PCR and thermocycler conditions are available in https://earthmicrobiome.org/protocols-and-standards/16s/. After DNA amplification, electrophoresis using 1.2% (wt/vol) agarose gels stained with 0.5 mg/mL ethidium bromide was used to verify amplicon presence and size. DNA purification was carried out using magnetic beads Mag-Bind TotalPure NGS (Omega Bio-Tek, Norcross, GA) in accordance with manufacturer instructions. Samples were standardized to the same concentration and pooled into a run for library preparation and sequencing, which was performed using the MiSeq Reagent Kit v2 (300 cycles) on the MiSeq platform (Illumina Inc.).

Non-biological nucleotides were removed, and raw sequenced amplicons were analyzed using the DADA2 package of RStudio Version 2023.06.1+524 (RStudio, PBC, Boston, MA) following the DADA2 Pipeline Tutorial (https://benjjneb.github.io/dada2/tutorial.html). After filtering and trimming, the amplicon sequence variant (ASV) table was constructed. Then, chimeric reads were removed, and the number of reads were standardized to the median read

number of all the samples. Taxonomy was assigned to ASV using the Greengenes database (http://greengenes.lbl.gov). More information about the procedures can be found in our previous study [17].

Total Bacteria 16S rRNA Gene Quantification

The total bacterial 16S rRNA gene quantification was carried out using the Femto™ Bacterial Quantification Kit (Zymo Research Corp, Irvine, CA) according to the manufacturer's instructions. First, DNA extracts were diluted to 1:10 prior to quantification. Briefly, 18 µL of the kit's master mix was added to each well with 2 µL of each sample. The PCR cycling condition consisted of 95 °C for 10 minutes for initial denaturation, 40 cycles of 95 °C for 30 seconds (denaturation), 50 °C for 30 seconds (annealing), and 72 °C for 1 minute (extension), followed by a final extension of 72 °C for 7 minutes. The amount of DNA in each sample was calculated based on the standard curve. Data for total 16S rRNA are described as nanograms of 16S rRNA per mL. All samples were run in duplicate. Intra-assay coefficient of variation for plates 1 to 8 were 1.01, 0.35, 1.34, 0.45, 2.80, 0.40, 0.28, and 0.61%, respectively. The inter-assay coefficient of variation was 0.91%. Estimated bacterial counts were calculated multiplying the total bacterial 16S rRNA by the relative abundance of each bacterial genus. Logarithms to the base 10 conversions of the raw values were then determined.

Uterine Metabolome Analysis

The second frozen uterine fluid aliquot was submitted to the University of California West Coast Metabolomics Center in Davis, CA for metabolome analysis. Samples were analyzed by technicians blinded to study groups using untargeted gas chromatography with time-of-flight mass spectrometry in a single batch as previously described [13, 14]. The carrier selected was helium gas, and a column comprised of 95% dimethyl/5diphenyl polysiloxanesne was used. The column flow rate was set at 1 mL/minute, and the initial oven temperature was set at 50 °C

followed by a 20 °C increase per min up to a final temperature of 330°C, which was held constant for a period of 5 minutes. Injection temperature was set to begin at 50 °C followed by a 12 °C increase per second up to 250 °C. Retention of primary metabolites was evaluated using default settings from ChromaTOF v. 2.32 and quantification was reported as peak height. Each metabolite was identified based on its mass and charge relationship. Metabolites were annotated using PubChem, Kyoto Encyclopedia of Genes and Genomes, and Human Metabolome Database. Of the 873 detected metabolites, a total of 253 metabolites were annotated, and 620 were unknown. Specific information about the metabolites can be found in our previous study [17].

Body Weight Data Collection

Primigravid cows were weighed twice at 240 d of gestation and the mean value was used as their prepartum body weight (ppBW). Multiparous cows' BW were collected from the on-farm computer software (Afifarm management program, Afimilk Ltd, Kibbutz Afikim, Israel). Three consecutive weights were collected at -2, -1, and 0 days relative to dry-off from the lactation preceding enrolment. The 3 measurements were used to generate a mean ppBW. On 0, 1, and 2 days relative to parturition weights were collected from the on-farm computer software from primiparous and multiparous cows. The 3 measurements were used to generate a mean calving BW (cBW). To only account for the body weight change (BWC) related to tissue accretion or mobilization, the weight of the gravid uterus prepartum and the weight of the empty uterus right after parturition were subtracted from the ppBW and cBW, respectively. The weight of the gravid uterus and empty uterus were calculated using NASEM (2021) [18] equations as follows:

$$Gravid\ Uterine\ Weight = \left[(Calf\ BW\ \times 1.825) \times e^{-[0.0243-(0.0000245\ \times day\ of\ gestation)]\ \times\ (280-day\ of\ gestation)}\right]$$

$$Empty\ Postpartum\ Uterine\ Weight = (Calf\ BW\ \times 0.2288)$$

After excluding the weight of the gravid uterus from the ppBW and the weight of the empty uterus from the cBW, the BWC prepartum was calculated as the difference between cBW and ppBW divided by the number of days between measurements. To account for any differences in BWC associated with frame, BWC then was calculated as a percentage of cBW.

References

1. NRC. Nutrient Requirements of Dairy Cattle. 2001.
2. Jeon SJ, Cunha F, Ma X, Martinez N, Vieira-Neto A, Daetz R, et al. Uterine microbiota and immune parameters associated with fever in dairy cows with metritis. PLoS One. 2016;11.
3. Casaro S, Marrero MG, Madrid DMC, Prim JG, Nelson CD, Galvão KN, et al. Flow cytometry panels for immunophenotyping dairy cattle peripheral blood leukocytes. Vet Immunol Immunopathol. 2022;248 March:110417.
4. Casaro S, Prim JG, Gonzalez TD, Bisinotto RS, Chebel RC, Marrero MG, et al. Unraveling the immune and metabolic changes associated with metritis in dairy cows. J Dairy Sci. 2023;106:9244–59.
5. Diez-Fraile A, Meyer E, Burvenich C. Regulation of adhesion molecules on circulating neutrophils during coliform mastitis and their possible immunomodulation with drugs. Vet Immunol Immunopathol. 2002;86:1–10.
6. Alhussien MN, Panda BSK, Kamboj A, Dang AK. Peripartum changes in the activity and expression of neutrophils may predispose to the postpartum occurrence of metritis in dairy cows. Research in Veterinary Science. 2021;135:456–68.
7. Xu H, Manivannan A, Crane I, Dawson R, Liversidge J. Critical but divergent roles for CD62L and CD44 in directing blood monocyte trafficking in vivo during inflammation. Blood. 2008;112:1166–74.
8. Rzeniewicz K, Newe A, Gallardo AR, Daviesa J, Holt MR, Patel A, et al. L-selectin shedding is activated specifically within transmigrating pseudopods of monocytes to regulate cell polarity in vitro. Proc Natl Acad Sci U S A. 2015;112:E1461–70.
9. Jakubzick C V., Randolph GJ, Henson PM. Monocyte differentiation and antigen-presenting functions. Nat Rev Immunol. 2017;17:349–62.
10. Kawai K, Tsuno NH, Matsuhashi M, Kitayama J, Osada T, Yamada J, et al. CD11b-mediated migratory property of peripheral blood B cells. Journal of Allergy and Clinical Immunology. 2005;116:192–7.
11. Morrison VL, Barr TA, Brown S, Gray D. TLR-Mediated Loss of CD62L Focuses B Cell Traffic to the Spleen during Salmonella typhimurium Infection . The Journal of Immunology. 2010;185:2737–46.

12. Galkina E, Tanousis K, Preece G, Tolaini M, Kioussis D, Florey O, et al. L-Selectin Shedding Does Not Regulate Constitutive T Cell Trafficking but Controls the Migration Pathways of Antigen-activated T Lymphocytes. Journal of Experimental Medicine. 2003;198:1323–35.
13. Fiehn O. Metabolomics by gas chromatography-mass spectrometry: Combined targeted and untargeted profiling. Curr Protoc Mol Biol. 2016;2016 April:1–32.
14. Fiehn O, Wohlgemuth G, Scholz M, Kind T, Lee DY, Lu Y, et al. Quality control for plant metabolomics: Reporting MSI-compliant studies. Plant Journal. 2008;53:691–704.
15. Sud M, Fahy E, Cotter D, Azam K, Vadivelu I, Burant C, et al. Metabolomics Workbench: An international repository for metabolomics data and metadata, metabolite standards, protocols, tutorials and training, and analysis tools. Nucleic Acids Res. 2016;44:D463–70.
16. Caporaso JG, Lauber CL, Walters WA, Berg-Lyons D, Huntley J, Fierer N, et al. Ultra-high-throughput microbial community analysis on the Illumina HiSeq and MiSeq platforms. ISME J. 2012;6:1621–4.
17. Casaro S, Prim JG, Gonzalez TD, Cunha F, Bisinotto RS, Chebel RC, et al. Integrating uterine microbiome and metabolome to advance the understanding of the uterine environment in dairy cows with metritis. Animal Microbiome 2024 6:1. 2024;6:1–13.
18. NASEM. Nutrient Requirements of Dairy Cattle: Eighth Revised Edition. NASEM. 2021. https://doi.org/10.17226/25806.
